# New-onset crescent IgA nephropathy following the CoronaVac vaccine: A case report

**DOI:** 10.1097/MD.0000000000030066

**Published:** 2022-08-19

**Authors:** Enrong Ran, Maohe Wang, Yanmei Wang, Rongzhi Liu, Yanxia Yi, Yuanjun Liu

**Affiliations:** a Department of Nephrology, Suining Central Hospital, Suining, China; b Department of Hepatobiliary Surgery, Suining Central Hospital, Suining, China.

**Keywords:** case report, COVID-19 vaccination, crescent IgA nephropathy, gross hematuria, kidney biopsy

## Abstract

**Rationale:**

Although coronavirus disease 2019 (COVID-19) remains a global threat, administering effective and safe vaccines is currently the most promising strategy to curb the ongoing pandemic and decrease the number of severe acute respiratory syndrome coronavirus 2 (SARS-CoV-2) infections. However, there remains some uncertainty regarding the safety of vaccines for patients with kidney disease.

**Patient concerns:**

A 58-year-old man presented at our institution with gross hematuria 48 hours after receiving his first dose of the CoronaVac (Sinovac) vaccine.

**Diagnoses:**

Analysis of a renal biopsy sample led to the diagnosis of crescentic immunoglobulin A nephropathy (IgAN), which we considered an adverse event of receiving the CoronaVac vaccine in China.

**Interventions:**

The patient’s serum creatinine and albumin levels were 1.20 mg/dL and 31.3 g/L, respectively; as such, he was administered a diuretic. His serum creatinine level had risen to 7.45 mg/dL 1 month later, and he developed high blood pressure. The patient then received conventional doses of hormone therapy but developed recurrent fever, which led to the suspicion of active tuberculosis (which he had a history of) and suspension of the hormone therapy.

**Outcomes:**

The patient’s renal function deteriorated further, and he ultimately underwent dialysis.

**Lessons:**

The patient’s course of events of apparent IgAN exacerbation should prompt nephrologists to closely follow patients with glomerular disease after they receive a COVID-19 vaccine, especially if persistent gross hematuria occurs.

## 1. Introduction

Ensuring effective and safe vaccines during the current coronavirus disease 2019 (COVID-19) pandemic is a global priority. However, there remain questions regarding the safety of currently available vaccines for patients with underlying conditions, including those with kidney disease. Herein, we report a patient with new-onset crescentic immunoglobulin A nephropathy (IgAN) following his receipt of the CoronaVac COVID-19 vaccine. This vaccine is based on an inactivated severe acute respiratory syndrome coronavirus 2 (SARS-CoV-2)^[1]^ and is produced by Sinovac Biotech, Beijing Institute of Biological Products Co., Ltd., China.

## 2. Case presentation

A 58-year-old man presented with gross hematuria along with generalized body aches and dizziness that developed 2 days after he received his first dose of the CoronaVac vaccine. He developed lower limb edema 5 days later, although no rash was observed. An outpatient examination at a local hospital revealed serum creatinine levels of 1.20 mg/dL (reference range: 0.46–1.00 mg/dL), albumin levels of 31.3 g/L (reference levels: 40–55 g/L), a urine protein score of 3+, urine occult blood score of 3+, and a 24 hours urine protein excretion amount of 1.69 g. The patient’s edema was relieved after the administration of diuretics; however, hematuria persisted. The patient had been hospitalized for tuberculosis 3 years prior. Proteinuria was not detected despite testing positive for occult blood in the urine. His serum creatinine level had been normal and no abnormality in renal function had been detected when he was administered anti-tuberculosis drugs. He had no history of hypertension or diabetes, nor did he have a family history of kidney disease.

**Figure 1.**
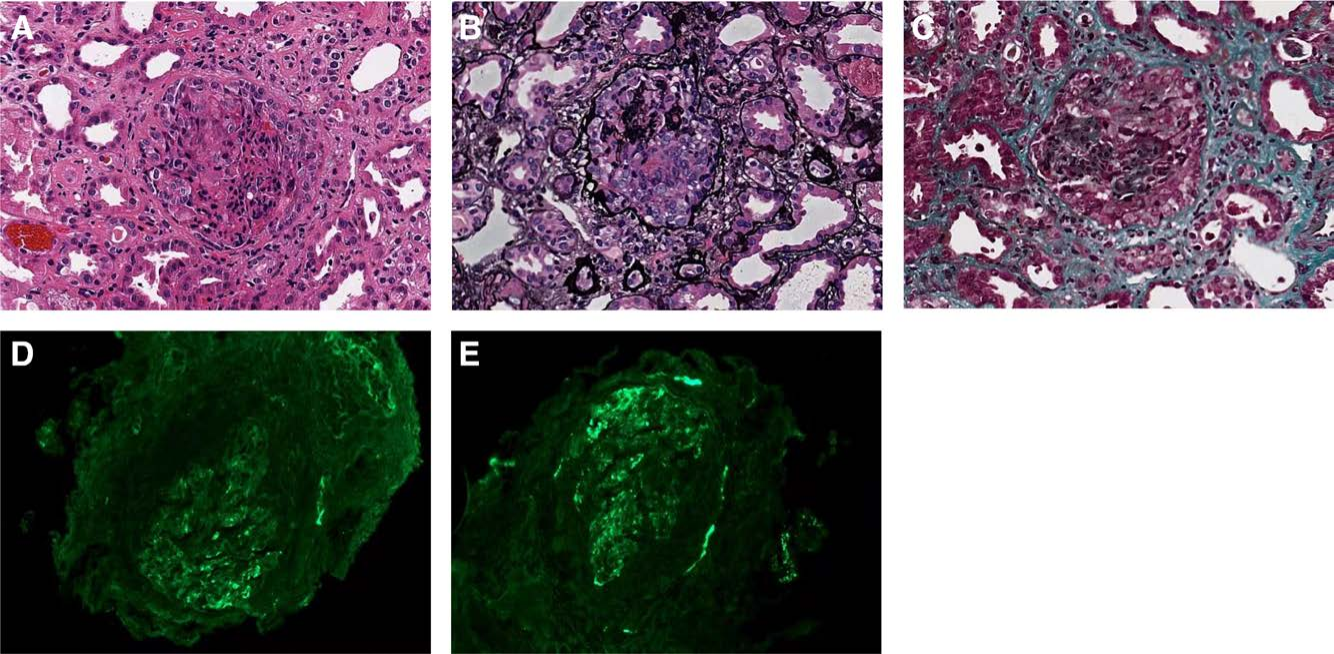
Kidney biopsy. Shown in the glomerulus stained with (A) hematoxylin-eosin (original magnification, ×200), (B) periodic acid-silver methenamine (original magnification, ×200), (C) Masson’s trichrome stain (original magnification, ×200), (D) anti-immunoglobulin A antibody (immunofluorescence; 2+), and (E) anti complement C3 (immunofluorescence; 2+).

He was admitted to our hospital 1 month after receiving the COVID-19 vaccine. Physical examination revealed a blood pressure of 190/100 mm Hg and mild edema in the lower extremities. However, he lacked other symptoms such as cough or fever. Laboratory tests revealed a serum urea level of 146.64 mg/dL, serum creatinine levels of 7.45 mg/dL, uric acid levels of 10.20 mg/dL, and albumin levels of 28.4 g/L. Urinary sedimentation revealed red blood cell levels of >100/high-powered field, a urine protein to creatinine ratio of 0.40, and a 24 hours urine protein excretion amount of 3.79 g. His liver function enzymes, blood glucose, thyroid hormone, immunoglobulin A (IgA) (4.21 g/L), and complement C3 and C4 levels were normal, and he was found to be negative for antineutrophil cytoplasmic antibody, anti-glomerular basement membrane antibody, anticardiolipase A2 antibody, and antinuclear antibody, as well as for hepatitis B, hepatitis C, and human immunodeficiency viruses. Chest computed tomography revealed a few nodules in both lungs with multiple calcified lesions observed in the mediastinum, hilar lymph nodes, and right pleura. Cardiac and renal ultrasonography showed no abnormalities. A renal biopsy revealed that, of the 11 glomeruli examined with light microscopy, 3 were globally sclerotic, 3 were cell crescents, 2 were cell fibrous crescents, 1 was a small cell crescent, and 1 was a small cell fibrous crescent (Fig. 1). Immunofluorescence revealed glomerular mesangial staining for IgA and complement C3; renal tubular epithelial cell granule and vacuolar degeneration were also observed. The patient received 40 mg qd of intravenous methylprednisolone; 1 week later, he developed recurrent fever and given that we could not rule out active tuberculosis or another pathogenic infection, methylprednisolone therapy was suspended. The patient’s renal function deteriorated further to the point that he underwent dialysis.

## 3. Discussion

Our patient was diagnosed with crescentic IgAN after presenting with gross hematuria, edema, hypertension, and a mild elevation of serum creatinine 48 hours after receiving the first dose of the CoronaVac (Sinovac) vaccine. The patient’s serum creatinine level increased to 7.45 mg/dL 1 month later, whereupon a kidney biopsy confirmed the diagnosis of crescentic IgAN. Since the patient had previously exhibited occult blood in the urine, he may have had IgA deposits in his kidney before undergoing vaccination, and thus likely had asymptomatic IgAN. However, whether the acute deterioration of renal function was coincidental or caused by the vaccination remains uncertain; no conclusive method of demonstrating a causal relationship currently exists.

We searched PubMed for all the literature describing patients who experienced IgAN or gross hematuria after receiving a COVID-19 vaccine published before December 31, 2021 ^[2–12]^ (Table 1), and extracted these patients’ baseline characteristics, clinical manifestations, and treatment and laboratory results. There were 25 patients, who were generally from the United States with a small number from France and Singapore, all received mRNA vaccination. Their median age was 38 (range, 13–67) years. Three patients were under 18 years, and the age of 2 patients was not reported.^[10]^ Thirteen of the 25 patients (52%) received the Moderna vaccine while the remaining 48% received the Pfizer-BioNTech Comirnaty^®^COVID-19 vaccine (BioNTech Manufacturing GmbH). To the best of our knowledge, our patient is the first to have experienced renal symptoms after receiving the CoronaVac vaccine from Sinovac. Nineteen patients from the literature (76%) presented with symptoms after the second dose of the vaccine, while 5 (20%) did so after the first dose; only 1 patient experienced symptoms twice after receiving each of the 2 doses. Gross hematuria was the most common symptom to occur following vaccination, along with varying degrees of proteinuria. Some patients with gross hematuria also presented with fever, body aches, headache, arthralgia, fatigue, and chills. Fourteen patients (56%) showed elevated serum creatinine levels, and 3 (12%) progressed to severe renal dysfunction. Seventeen of the 25 patients (68%) received only conservative treatment (including angiotensin-converting enzyme inhibitors) and 8 (32%) received hormone therapy; among the latter, one underwent plasma exchange while another (a pediatric patient) received high-dose hormone therapy and underwent temporary hemodialysis after developing rapid renal function deterioration and oliguria.

**Table 1:** Summary of clinical features of the patients with gross hematuria after receiving COVID-19 vaccine.

Study	Age/sex	Country	Comorbidities	New onset or relapse	Types of vaccine	Vaccine dose	Onset time (D, W)	Creatinine (mg/dL)		Biopsy	24 h urine protein (g/d) {uPCR [g/g]}		Treatment
								Presentation	Follow up		Presentation	Follow up	
Tan et al^[11]^	41/F	SG	GDM	N	mRNA (Pfizer)	2	D2	1.73	-	Yes	{2.03}	-	Steroid + CTX
Tan et al^[11]^	60/F	SG	HPL	N	mRNA (Pfizer)	2	D2	6.12	-	Yes	{7.58}	-	Steroid + CTX + PE
Hanna et al^[4]^	17/M	US	No	N	mRNA (Pfizer)	2	D1	1.78	1.20	Yes	{1.75}	{1.20}	Steroid
Kudose et al^[3]^	50/F	US	HTN, obesity, APS	N	mRNA (Moderna)	2	D2	1.70	-	Yes	{2.00}	-	-
Kudose et al^[3]^	19/M	US	No	N	mRNA (Moderna)	2	D2	1.20	-	Yes	-	-	-
Park et al^[5]^	22/F	US	IgA vasculitis	N	mRNA (Moderna)	2	D2	0.80	0.80	No	0.4	0.27	Conservative
Park et al^[5]^	39/F	US	No	N	mRNA (Moderna)	2	D2	0.80	0.80	No	0.9	-	Conservative
Park et al^[5]^	50/M	US	HTN	N	mRNA (Moderna)	2	D1	1.54	1.24	Yes	3.56	2.2	Conservative
Park et al^[5]^	67/M	US	HTN	N	mRNA (Moderna)	1	M1	2.90	1.40	Yes	2.1	0.09	Steroid
Abramson et al^[6]^	30/M	US	No	N	mRNA (Moderna)	2	D1	1.02	1.03	Yes	{0.80}	{0.43}	Conservative
Klomjit et al^[8]^	38/M	US	No	N	mRNA (Pfizer)	2	2W	1.60	-	Yes	0.32	-	Conservative
Klomjit et al^[8]^	44/M	US	No	N	mRNA (Moderna)	1	2W	2.50	3.60	Yes	14 g	5.6	Steroid
Klomjit et al^[8]^	66/M	US	No	N	mRNA (Moderna)	1	2W	1.50	1.40	Yes	1.2 g	0.3	Steroid
Klomjit et al^[8]^	62/M	US	No	N	mRNA (Pfizer)	2	6W	2.20	2.00	Yes	0.9 g	0.2	Conservative
Niel and Florescu^[12]^	13/F	LU	No	N	mRNA (Pfizer)	1	D1	3.57	0.86	Yes	3.88	-	Steroid + HD
Hanna et al^[4]^	13/M	US	IgAN, T1DM	R	mRNA (Pfizer)	2	D1	1.31	0.66	No	{1.07}	{0.86}	Conservative
Rahim et al^[7]^	52/F	US	IgAN	R	mRNA (Pfizer)	2	D1	0.80	0.80	No	2.41	1.44	Conservative
Plasse et al^[10]^	-	US	IgAN	R	mRNA (Pfizer)	2	D1	3.53	Baseline	No	3	Baseline	Steroid
Plasse et al^[10]^	-	US	IgAN	R	mRNA (Pfizer)	2	D1	1.16	-	No	0.92	-	Conservative
Negrea and Rovin^[2]^	38/F	US	IgAN	R	mRNA (Moderna)	2	D1	-	-	No	0.82	1.4	Conservative
Negrea and Rovin^[2]^	38/F	US	IgAN	R	mRNA (Moderna)	2	D1	-	-	No	0.59	0.4	Conservative
Perrin et al^[9]^	22/M	FRA	IgA vasculitis	R	mRNA (Moderna)	1, 2	D2, 25	0.40	0.4	No	0.34	0.4	Conservative
Perrin et al^[9]^	41/F	FRA	IgAN-Grafting	R	mRNA (Pfizer)	1	D2	-	-	No	0.47	0.41	Conservative
Perrin et al^[9]^	27/F	FRA	IgA -HD	R	mRNA (Pfizer)	2	D2	HD	HD	No	1.9	1.2	Conservative
Klomjit et al^[8]^	19/M	US	IgAN	R	mRNA (Moderna)	2	1W	0.76	-	No	0.61	-	Conservative

APS = antiphospholipid syndrome, CTX = cyclophosphamide, D = day, Dose = which dose of COVID-19 was injected, F = female, GDM = gestational diabetes, HD = hemodialysis, HPL = hyperlipidemia, HTN = hypertension, IgAN = IgA nephropathy, M = male, PE = plasma exchange, TB = tuberculosis, T1DM = type 1 diabetes, W = week.

Patients who received conservative treatment experienced relief of their gross hematuria within 1 week. Steroid treatment was effective in patients with a mild-to-moderate elevation of serum creatinine levels except for a single individual^[11]^ who did not seek medical attention immediately after developing gross hematuria; this patient’s serum creatinine level was >6.0 mg/dL 1 month later, and we speculated that the delay in treatment was the main reason for this deterioration in renal function.

Thirteen patients are reported in the literature to have experienced new-onset IgAN after receiving a COVID-19 vaccine, as are another 10 who relapsed. Of the former, 11 were diagnosed via renal biopsy while 2 patients who presented with gross hematuria following their vaccinations were presumed to have IgAN despite a lack of biopsy. None of the latter 10 patients underwent biopsies as they had previously confirmed IgAN; among these relapsed patients, one undergoing maintenance hemodialysis also developed gross hematuria, while another did so after renal transplantation.^[9]^

A previous systematic review found that minimal change disease (MCD) as the most frequent pathology observed after COVID-19 vaccination, while IgAN and vasculitis are subsequently diagnosed.^[13]^ Lim et al’s^[14]^ report suggested that MCD usually occurred within 10 days after receiving the first dose of vaccine, whereas we found that new onset or exacerbated IgAN mainly occurred 24 to 48 hours after the second dose of mRNA vaccine. Although the exact pathogenesis is still unelucidated, the increased proportion of circulating CD4+ T cells, CD8+ T cells, and neutralizing antibodies is thought to have differing roles.^[15]^ Vaccination-related nephrotic injury associated with the administration of other vaccines,^[16]^ such as for influenza, hepatitis B, pneumococcus, and measles, has also been reported; MCD and acute kidney injury occurred more frequently, while IgAN was less common.

Although we described a potential association between COVID-19 vaccination and the onset or exacerbation of IgAN in our current literature review, no conclusive means to demonstrate causality currently exist; our evidence is based on timing and relies on the exclusion of other factors. Most of the patients we reviewed had a good prognosis. Vaccination reportedly induces robust T cell activation and cytokine release along with strong antibody responses, which might have contributed to the crescentic IgAN in our patient. T cell responses are evident after the first dose of the Pfizer-BioNTech vaccine, and a substantial increase in their magnitude is observed after the second dose,^[17]^ this may be why new-onset or exacerbated IgAN more frequently occurred after the second dose. Large-scale vaccination is a promising way to end the COVID-19 pandemic, although whether certain types of vaccines are better than others remains unclear. Since IgAN is more common in the Chinese population, more clinical trial data are needed to fully assess the possible correlation between IgAN and COVID-19 vaccination. The reported episodes of apparent IgAN exacerbation should prompt the nephrology patient care community to closely follow patients with glomerular disease after they receive a COVID-19 vaccine, especially if persistent gross hematuria occurs.

## Acknowledgments

We thank Editage (www.editage.cn) for English language editing.

## Author contributions

Conceptualization: Enrong Ran and Yuanjun Liu; Writing—original draft: Enrong Ran; Writing—review & editing: Enrong Ran, Maohe Wang, Yanmei Wang, Rongzhi Liu, Yanxia Yi, and Yuanjun Liu.
